# The role of nitrogen in achieving sustainable food systems for healthy diets

**DOI:** 10.1016/j.gfs.2020.100408

**Published:** 2021-03

**Authors:** Adrian Leip, Benjamin Leon Bodirsky, Susanna Kugelberg

**Affiliations:** a European Commission, Joint Research Centre, Ispra, VA, Italy; b Potsdam Institute for Climate Impact Research (PIK), Potsdam, Germany; c Public Health Consultant, Copenhagen, Denmark

## Abstract

The ‘food system’ urgently needs a sustainable transformation. Two major challenges have to be solved: the food system has to provide food security with healthy, accessible, affordable, safe and diverse food for all, and it has to do so within the safe operating space of the planetary boundaries, where the pollution from reactive nitrogen turned out to be the largest bottleneck. Here we argue that thinking strategically about how to balance nitrogen flows throughout the food system will make current food systems more resilient and robust. Looking from a material and a governance perspective on the food system, we highlight major nitrogen losses and policy blind spots originating from a compartmentalization of food system spheres. We conclude that a participatory and integrated approach to manage nitrogen flows throughout the food system is necessary to stay within regional and global nitrogen boundaries, and will additionally provide synergies with a sustainable and healthy diet for all.

## Highlights

• Mitigating nitrogen pollution requires a food system approach.
• A nitrogen perspective helps embark on the right road towards food system transformation.
• Food and nitrogen system governance has to target food processing and retail industries.

## 1 Introduction

“A food system gathers all the elements (environment, people, inputs, processes, infrastructures, institutions, etc.) and activities that relate to the production, processing, distribution, preparation and consumption of food, and the outputs of these activities, including socio-economic and environmental outcomes. […].” (HLPE, 2017).

Nitrogen is an essential building block for the whole food system from farm to mouth. For food production, both under- or oversupply of nutrients are problematic. An unbalanced nitrogen cycle causes an environmentally unsustainable food system (Rockström et al., 2020; W. Steffen et al., 2015b), resulting in air and water pollution, affecting soil quality, and contributing to climate change or biodiversity loss (Leip et al., 2015; Sutton et al., 2011). Nitrogen budgets involve all anthropogenic activities that produce, transfer, emit or receive reactive nitrogen along the ‘nitrogen cascade’ (Galloway et al., 2003), including the environmental, human, or socio-cultural impact caused by reactive nitrogen losses (see glossary). Transforming the food system to align with critical thresholds for reactive nitrogen pollution has been identified as the most restricting planetary boundary in integrated modelling studies (Gerten et al., 2020; Springmann et al., 2018).

A required transition towards healthy and sustainable diets is acknowledged in multiple studies such as the EAT-Lancet report (Rockström et al., 2020; Willett et al., 2019). There are multiple synergies from combining nitrogen and food system research, understanding common drivers and bottlenecks and to better identify “win-win” versus “win-loose” scenarios for integrated policy recommendations.

While the role of nitrogen has been thoroughly analyzed in different food system spheres, e.g. during food production and farming, with the main objective to increase food productivity and security, there have, surprisingly, been very few attempts to understand the dynamics of nitrogen flows throughout the food system and with the aim to achieve a sustainable food system transformation. In addition, the climate change perspective on food systems is very prominent in policy and research communities (Harwatt et al., 2019), but there have been much less investigations in respect to nitrogen pollution even though the impacts are of comparable magnitude (Leip et al., 2011a; Will Steffen et al., 2015a; Sutton et al., 2011, 2013).

Hence, this special issue provides a nitrogen perspective on food systems. This particular perspective doesn't intend to narrow down the understanding of complex food systems, nor is the intention to subordinate the manifold functions of food systems below the aim of reducing nitrogen pollution. Rather, this special issue raises to the challenge to highlight the crosscutting theme of nitrogen across the food system spheres, and the multiple synergies between closing the nitrogen cycle and transforming our current dysfunctional food system towards a healthy and sustainable food system.

## 2 Narrowing the knowledge gap on the ‘nitrogen and food’ systems

The papers within this special issue show how nitrogen is a linking element within the food system, and how central it can be for the understanding of the food system. They approach the topic from several disciplinary backgrounds, ranging from agronomy, nutrition to political science and economics. And they look at the different spheres of the food system, such as agriculture, food supply chain, or food consumption.

Hutchings et al. (2020, this issue) created a new model to analyze consistently how farm level mitigation measures may change multiple nitrogen losses and reduce the risk of pollution-swapping. Corrado et al. (2020, this issue) look at the reduction of food waste as important link between food systems and the circular economy strategies. Weindl et al. (2020, this issue) discuss the role of nitrogen in a healthy diet. In the form of amino acids, nitrogen is a major constituent of food, and an adequate supply of amino acids (in terms of quality, quantity and combination) is an important element in the definition of a healthy diet (Hoffer, 2014; Sonesson et al., 2017; Weindl et al., 2020, this issue). Weindl et al. (2020, this issue) show that there is abundant literature on single proteins but scarcely on their interactions, yet diets themselves are systems that are more than the sum of the ingredients.

Foods cover a large range of nitrogen footprints with strong variation across regions and production system, but emissions from meat, in particular ruminant meat consistently rank highest (Gu et al., 2013; Hutton et al., 2017; Leach et al., 2012; Leip et al., 2014; Leip and Uwizeye, 2019; Liang et al., 2016; Pierer et al., 2014; Poore and Nemecek, 2018), and diets with limited meat consumption have been shown to reduce nitrogen pollution (Springmann et al., 2018). A review of different diets concluded that vegan diets may reduce N and GHG footprints by about 50% as compared to current average diets (Sanz-Cobena et al., 2020). While on average, the largest protein source in Europe comes from livestock production, more and more people are choosing diets with predominantly vegetable protein sources. Plant-based diets are not healthy *per se* but both plant-based and omnivorous diets can be planned balanced and healthy. This also holds true for vegan diets under most circumstances, if precautions are taken to ensure sufficient Vitamin B12 intake (Costa Leite et al., 2020, this issue). Yet, even though a few national food based dietary guidelines (e.g. Brazil and Qatar) or government dietary advice (e.g. Germany, Netherlands, Sweden and the UK, Lang and Mason, 2018) have taken up sustainability aspects, guidance and policy support is so far generally missing for healthy individuals choosing such dietary patterns.

Consumers' food choices are largely determined by the food environment in their vicinity (HLPE, 2017, see also Glossary). Emerging technologies such as urban and controlled environment agriculture have the potential to resolve some bottlenecks that exist in current food systems, such as low consumption of fruit and vegetables, and supply urban centers with fresh and local products with low environmental footprints throughout the year, if based on renewable energy sources (Armanda et al., 2019; Benke and Tomkins, 2017; O'Sullivan et al., 2019). Policies and measures targeting the food environment are increasingly being implemented since the last decade, but have so far been moderately effective, and include the implementation of nutrition labelling, fiscal policies, *trans*-fat bans, reformulation of food products, and restricting marketing of foods and non-alcoholic beverages to children (WHO, 2018). So far, these policies are mainly in place for public health nutrition concerns, but could be used to promote dietary change towards a healthy *and sustainable* diets, aligned with N reduction strategies (Temme et al. 2020).

Dietary shift is often seen as a matter of individual choice, yet there is a risk to put too much responsibility on the ‘power of the consumer’ which is often limited (Group of Chief Scientific Advisors, 2020; Temme et al. 2020). Projects requiring active participation such as in urban gardening can help to change food habits, as has been shown by Puigdueta et al. (2019) on the example of Madrid. If transition to a more healthy diet were to be achieved by taxes, simulations show that they would need to be prohibitively high (Latka et al. 2020 n. d., this issue). Sustainable food systems therefore cannot be achieved by single measures; they need a deep transformation that cover all spheres of the food system, including the political sphere (Kugelberg et al. 2020, this issue).

Assessing the overall sustainability of a product is multi-dimensional and complex, and no agreed methodology exists so far that integrate very different aspects such as animal welfare, fair trading praxis, or greenhouse gas emissions. A comprehensive metrics framework that could support not only consumer information but also integrated national food policies and assess potential synergies and trade-offs is therefore urgently required (Brouwer et al., 2020). System change from status quo to an integrated food system policy approach need further attention to building up a policy-making process that provide a strong directionality towards sustainability (and not only economic growth) and enable a greater reflexivity of the policy cycle. This can be facilitated from a participatory and integrated vision-building processes, built on the engagement of marginalized voices and lessons learnt from integrated food system metrics and evaluations produced by state and non-state actors (Kugelberg et al. 2020, this issue).

## 3 Food systems from a nitrogen perspective

**Fig. 1:**
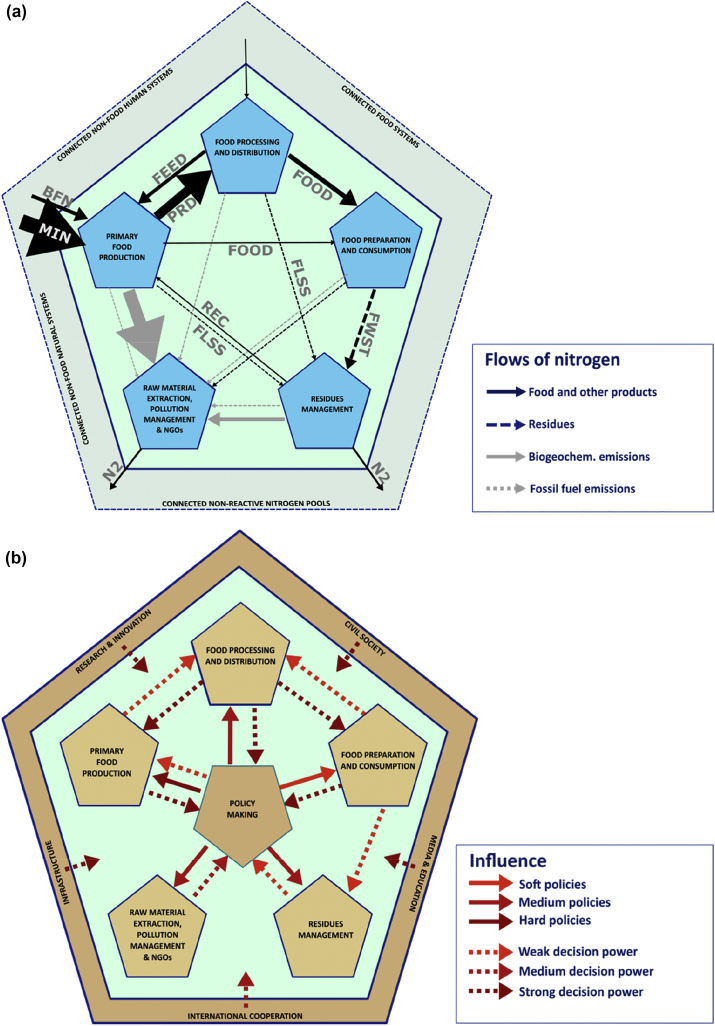
Conceptual framework of the material (nitrogen) and governance (relations and decision-making power) aspects of food systems. a) Nitrogen flows between the five EU food system spheres (blue pentagons) and across the EU food system boundaries to connected food (e.g. a food system from a different country or region where some functions are carried out by the same actors, see glossary) and non-food systems. Widths of arrows are proportional to the N flow rate (Corrado et al., 2020, this issue; Leip et al., 2015, 2014), with minimum arrow size corresponding to 1 Mt N yr^−1^. Black arrows: intended flows of mineral fertilizer (MIN); biological nitrogen fixation (BFN); agricultural products (PRD); feed returned to agriculture (FEED); nutrient recovery (REC); food (FOOD); flows of food residues as food loss (FLSS) and food waste (FWST). Grey arrows: unintended emissions of reactive nitrogen from biogeochemical processes or fossil fuel. b) Governance aspects of food system spheres (orange pentagons). Influences exerted from and on food system governance actors in the EU food system are indicated with red arrows, using solid lines for governmental regulations and policies, and dotted lines for other influences (decision-making power). Influences are also exerted from other relevant elements of social setting indicated in the outer orange pentagon; influences between these groups and food system actors are manifold and not comprehensively shown. (For interpretation of the references to colour in this figure legend, the reader is referred to the Web version of this article.)

To rethink food system from a nitrogen perspective, we introduce here a conceptual framework (see Fig. 1) to highlight two different perspectives on the food system and its subsystems, the “food system spheres” (see glossary): the material perspective and the governance perspective.

### 3.1 A material perspective on nitrogen in food systems

The ‘*Material perspective'* looks at material stocks (e.g. biomass or nitrogen), flows (between one food system sphere and another, or cross the boundaries of the food system to flow into/out of another food or non-food system), transformations (e.g. from ammonia to a complex protein) and biophysical impacts (e.g. of diets on the probability to develop cancer) that are present in food systems, and connect one food system sphere with another. Fig. 1a illustrates this with the flow of nitrogen in the EU food system (data from Corrado et al., this issue and Leip et al., 2015).

Looking with a material perspective on food systems shows that nitrogen is a cross-cutting material within many spheres of the food system:

In some parts of the low-income countries there is limitation of nitrogen with severe consequences for food security and environmental degradation (Bekunda et al., 2010; Hutton et al., 2017; Sanchez et al., 2007; Sánchez, 2010). However, in Europe and in most middle and high-income countries, supply of nitrogen in agriculture is not limited and problems are linked to losses of nitrogen to the environment (Galloway et al., 2013; Leip et al., 2011b; Westhoek et al., 2014). Consequently, a major part of nitrogen emissions is associated with food production, a significant share of which producing food that is wasted (Caldeira et al., 2019; WFP et al., 2019; Verma et al., 2020; FAO, 2019). For Europe, the share of food-related nitrogen emissions was estimated at 94% for NH_3_ emissions or 55% for total NH_3_ and NO_x_ emissions, and 59% to water pollution with N (Leip et al., 2015). These numbers refer only to cradle-to-farm gate emissions and exclude emissions from feed imports; which are estimated to account for 39% of total agricultural GHG emissions, as well as 11% of land use or 8% of total agricultural NH_3_ emissions (Leip et al., 2015).

Production of animal proteins disproportionally contributed to most of the environmental problems related to nitrogen (Behrens et al., 2017; Leip et al., 2015; Sanz-Cobena et al., 2020; Springmann et al., 2018). Ruminant supply chains are responsible for roughly 10% of global GHG emissions (Gerber et al., 2013), and livestock also has a dominant role for further environmental problems (Leip et al., 2015; Steinfeld et al., 2006). At the same time, the consumption of red meat exceeds the healthy recommendations in many countries (Behrens et al., 2017; Clark et al., 2018, 2019). High consumption of red and processed meat are associated with several types of cancers, but perhaps more importantly, the high consumption of animal products replaces other, healthier food groups, e.g. legumes, nuts, fruits and vegetables (GBD 2017 Diet Collaborators, 2019; 2018). Animal welfare concerns also add to an increasing number of people asking for diets reduced in animal products (Scherer et al., 2018), and the debate is obviously also fueled by the climate emergency (Ripple et al., 2019). Undernourishment and hunger co-exist with the increase of non-communicable diseases (NCDs), overweight and obesity due to malnourishment and over-consumption of food. This is true globally, yet is also found within individual countries (FAO et al., 2020). Technology can solve parts of the environmental problems of nitrogen, but for serious improvements (UNEP, 2019) we need to restructure the entire food system to reach a sustainable state (Ingram, 2017; IPES-Food, 2019; Rockström et al., 2020).

### 3.2 A governance perspective on nitrogen in food systems

A ‘*governance perspective’* on food systems shows food system actors' relative control and power over resources and core functions, including those in other food system spheres (see glossary). Food system governance includes both state and non-state actions influencing the food system, and is taking place in governments, private companies, civil society organizations, or individual citizen (Voβ and Kemp, 2006) (see glossary for definition of food system functions, actors, stages, boundaries).

Fig. 1b illustrated the distribution of power within EU food system spheres. Primary producers, food processors and distributors, and consumer are all mutually - but not equally - influencing food supply and demand, N flows and transformations, and other food system outcomes e.g. on the environment or economy (Zurek et al., 2018). To a certain extent, consumers influence what products are offered in the retail and food services, and indirectly influence farming decisions. However, compared to business and private sector actors working in the food supply chain, consumers have relatively low influence and are poorly organized to drive policy changes and achieve the healthy and sustainable food system they might wish for (Broers et al., 2017; Bucher et al., 2016; Group of Chief Scientific Advisors, 2020; Tørris and Mobekk, 2019). This is to some extent also true for the primary producers. Decisions affecting the food supply chain are to a large extent dominated by the food processing and distribution spheres who shape the external food environment, and influence consumer choices partly through aggressive marketing and nudging strategies (Clapp, 2019; Group of Chief Scientific Advisors, 2020; Howard, 2016). Simultaneously, the monopsonic position of food processors and distributors allows them to largely control the primary production sector, the agricultural producers (Fałkowski et al., 2017).

Transforming the food system will require not one single policy instrument, but the combination of multiple instruments (Swinburn, 2008). Hence a mix of innovative, disruptive and established policies (Kanter et al., 2020). The CAP distributes the largest share of the EU budget, but has been criticized by not making sufficient use of its potential to steer the food system towards a healthy and sustainable food system (Pe'er et al., 2019). Other policies address the food processing and distribution sphere include voluntary and mandatory food reformulation, standards and labelling (Mozaffarian et al., 2018; Niebylski et al., 2015), and regulations on supermarket food waste (Albizzati et al., 2019). Those policies are motivated by public health goals, e.g. to combat child obesity. Examples are obligatory labelling for products high in saturated fats, salt or sugar obesity (such as in Chile, Corvalán et al., 2019) or the green keyhole in the Nordic countries to positively guide consumers to healthy food choices high in fiber but low in fat, sugar and sodium (Larsson et al., 1999). Further policies include taxes on junk food, fat and sugar (Apostolidis and McLeay, 2016), public food provision in canteens of schools, universities or the military, or even altering the obesogenic food environments (Swinburn, 2008), e.g. by expanding bike lanes for active transportation, or by creating advertisement free zones.

### 3.3 Integrating both perspectives on nitrogen in food systems

Food system policy has traditionally been compartmentalized to different policy areas, such as agriculture, food security, public health or competitiveness & innovation. Each policy sector is under the responsibility of different ministries and agencies, which communicate, regulate and manage the functions, outputs and actors within their respective sector. Some policy issues, such as air quality policy or wastewater policy have been treated as disjoint from the food system. A system approach however shows that these policy areas are connected both by material flows and by actors’ activities (Fig. 1), and therefore would benefit from policy coordination and a collaborative governance frameworks (Sutton et al., 2019; UNEP, 2019).

Eutrophic nitrogen in a lake may have travelled along the entire food supply chain, from its initial fixation in a fertilizer plant, through croplands, several loops in the animal or processing sector, until it reached human consumption and was flushed into wastewater systems. Moreover, after it reached natural systems, it may simultaneously or subsequently create multiple impacts across environmental areas, the so-called nitrogen cascade (Galloway et al., 2003). This suggests a collective responsibility of multiple actors across the food supply chain to solve the multiple nitrogen problems, independently of different spatial and temporal scale of actions and effects.

Undernourishment, diet-related non-communicable diseases and environmental pollution should not be analyzed and managed separately in academic and policy-making silos, but should be recognized as different symptoms of a dysfunctional food system (Nature Food, 2020; Swinburn et al., 2019). These symptoms are heavily intertwined, share common drivers and require coordinated solutions. If we solve the nitrogen problem, food systems will already improve; better nitrogen management can reduce environmental pressure, help to increase productivity in developing countries and contribute in moving people out of food scarcity.

Therefore, a multi-level governance across the food system is required to effectively and efficiently solve problems such as nitrogen pollution or malnutrition. Collaboration and coordination is needed at and across multiple levels (global, regional and local) and should be led by trusted authorities. To achieve transformative change, it is essential to rethink current policy frameworks. Policy change can be facilitated by participatory policy-making processes that engages all actors giving equal ground for both dominant and marginalized actors, and involving societal and business actors, to draw lessons from contexts, practices and norms. This process should be supported by independent research pointing out major policy gaps on, and possible solutions to, managing N losses and flows within food systems, highlighting consequences of action and important trade-offs between policy goals to decision makers (Kugelberg et al. 2020, this issue), to guard against the risk of ignoring large potential of synergetic or antagonistic effects.

## 4 Towards integrated solutions

But how does an integrated and governance approach to the ‘nitrogen and food system’ help to mitigate nitrogen pollution, or contribute in making the food system transition towards sustainability happen?

From a nitrogen perspective the answer is rather straightforward: any measure that reduces food demand, foremost for livestock products, helps reducing losses of reactive nitrogen to the environment along the entire food supply chain. Several studies show that technological improvements and more efficient production management alone are insufficient to reduce the environmental pressure to a sustainable level (Bodirsky et al., 2014; Gerten et al., 2020; Springmann et al., 2018). Hutchings and colleagues (Hutchings et al., 2020, this issue) have calculated that even with ambitious environmental targets, currently available technologies will sufficiently reduce farm-level losses of reactive nitrogen. On the other hand, policy instruments targeting the diet of Europe's citizen can be powerful instruments in achieving nitrogen reduction targets (Chai et al., 2019; Sanz-Cobena et al., 2020).

An integrated narrative for achieving sustainability targets is gaining a common ground which emphasizes the need of a combined effort on the supply and demand side, (Clark et al., 2019; Gerten et al., 2020; Gil et al., 2019; IPES-Food, 2019; Mbow et al., 2019; Rockström et al., 2020; Rosenzweig et al., 2020; Springmann et al., 2018; Willett et al., 2019). Bottom-up city initiatives such as the Milan Urban Food Policy Pact aim to take such an integrated approach to food policy (Candel, 2019). On a European level, the European Commission launched the ‘European Green Deal’ (European Commission, 2019), that, amongst others, aims to transform the European food system into one that is ‘fair, healthy, and environmentally friendly’, a task that has been concretized in the ‘Farm to Fork Strategy’. Also national (European Commission, 2020). Also, national strategies for integrated food system policies are emerging (e.g. Sweden, Finland, UK, France, Canada, South Africa or Australia; Carey et al., 2016; Kugelberg et al. 2020 n. d., this issue; Parsons, 2018; Termeer et al., 2018). While these national policies show a good political intention, they reflect only incremental changes and don't rise to challenge the dominant food production paradigm.

A shift in demand at the required scale is unlikely to happen unless policies to shift dietary intake to a sustainable diet are also supported by policies from other sectors, e.g. social, economic, trade, to address important key determinants in the food environment affecting food choices.

## 5 Conclusion

An effective nitrogen policy necessitates a holistic view on environmental and circularity aspects. On the one hand, policies promoting sustainable and healthy diets, environmental sustainability and circularity thinking provide synergetic effects on reducing N pollution, on the other hand conventional food production and trade policies pose a huge risk for engraving N's planetary boundary. Hence, from a nitrogen perspective, this paper claims that only serious efforts to develop a holistic and integrated food system policy, will succeed in keeping N within its safe operating space.

Looking at food systems from a wider sustainability perspective, a food system strategy with a nitrogen lens and with ambitious pollution reduction targets in mind will almost automatically deliver co-benefits in the context of sustainable development.

## Declaration of competing interest

The authors declare that they have no known competing financial interests or personal relationships that could have appeared to influence the work reported in this paper.

## Figures and Tables

**Table:** 

Food system	“A food system gathers all the elements (environment, people, inputs, processes, infrastructures, institutions, etc.) and activities that relate to the production, processing, distribution, preparation and consumption of food, and the outputs of these activities, including socio-economic and environmental outcomes. […].” (HLPE, 2017).
Nitrogen budgets	Nitrogen budgets involve all anthropogenic activities that produce, transfer, emit or receive reactive nitrogen along the ‘nitrogen cascade’, including the environmental, human, or socio-cultural impact caused by reactive nitrogen losses. (See (UN-ECE, 2010))
Food environment	“The food environment refers to the physical, economic, political and socio-cultural context in which consumers engage with the food system to acquire, prepare and consume food. The food environment consists of: “food entry points”, i.e. the physical spaces where food is obtained; the built environment that allows consumers to access these spaces; personal determinants of food choices (including income, education, values, skills, etc.); and the political, social and cultural norms that underlie these interactions. The key elements of the food environment that influence food choices, food acceptability and diets are: physical and economic access to food (proximity and affordability); food promotion, advertising and information; and food quality and safety” (HLPE, 2017).
Food system functions*	A *food system function* is any activity, service or statement that is part of or directly influences a food system. Food system functions include
	- production or transformation of a product, residue, or waste that is part of a food system, including extraction of raw materials, and food ingestion
	- storage, transportation or trading of a product, residue, or waste of a food system
	- providing advice or other non-material services that affect the functioning of the food system, including marketing or anything else that directly influences choices made
	- exerting management or control functions, such as policies regulations, information, etc.
Food system actors**	*Food system actors* are all persons that carry out any of the food system functions. A single person can carry out one or several food system functions, for example does every person consume food. The food system actor makes choices how to carry out the functions (e.g. what to eat, how to manage the farm, …). Choices of food consumption are influenced by the external and internal *food environment*. Not necessarily are decisions consistent a food system actor makes for different functions. It is therefore often more appropriate to work with food system functions; ‘the retailer’ therefore usually refers to the function of a person to sell goods (e.g. meat), even if the same person as ‘consumer’ could follow an ethical vegetarian diet.
Food chain**	*Food chains* include all food system functions contributing to the production and provision of a food product from extraction of raw material to consumption in its final form, including materials and services used for processing, distribution, storage and packaging.
	The concept of *food supply chains* focuses on the physical flow of materials through the food chain, while *food value chains* include also functions that do not necessarily displace or transform a product but add value.
Food consumption**	Food is consumed in its final distribution step before it is ingested or prepared for ingestion. For example, citizen consume food when they buy it in the supermarket, or a restaurant consumes food when it is delivered to it. Between food consumption and food ingestion, food can be transported, prepared, served; food product residues (from the food itself or from packaging) can be separated and transferred to food residues management systems.
Food system stages**	Food and other material used in a food system move along food supply or food value chains from raw materials to the final food product and the management of food residues and wastes. *Food system stages* bundle different *food system functions:*
	- provision of raw materials and inputs required in subsequent food system stages, such as fertilizers and agro-chemicals, machinery, plastics and other packaging materials etc.
	- primary food production (food commodities),
	- food processing (from food commodities to food products, including food commodity trading),
	- distribution (including packaging, storage, transportation and retail),
	- consumption (including catering and food preparation and ingestion),
	- end-of-life (including food residues management and management of non-food residues that had been used in pervious food system stages)
Material and governance perspectives of food system spheres*	*Food system spheres* allow to look at food sub-systems from different perspectives. A ‘*material perspective*’ on food systems looks at material stocks (e.g. biomass or nitrogen), flows (between one food system sphere and another, or cross the boundaries of the food system to flow into/out of another food or non-food system), transformations (e.g. from ammonia to a complex protein) and biophysical impacts (e.g. of diets on the probability to develop cancer) that are present in food systems.
	A ‘*governance perspective’* on food systems shows food system actors' relative control and power over resources and core functions, including those in other food system spheres. Food system governance includes both institutional and non-institutionalized actions influencing the food system and taking place in governments, research, private companies, civil society organizations, or individual citizen (Voβ and Kemp, 2006) (see definition of food system functions, actors, stages, boundaries). Conceptually, food system spheres align with food system stages with regard to the *material perspective*, combining the food processing and food consumption stage:
	- *Environment*: delivers raw material and other resources (land, water, fossil fuels, etc.) and receives waste materials (organic and non-organic wastes and chemical wastes/pollutants).
	- *Primary food production:* produces plant, animal, and microbial products that are partly or fully destined for human nutrition. The primary food production sphere includes all industries producing inputs required for food production, such as fertilizers, agro-chemicals, machinery. The environment and primary food production spheres contain the information required for ‘cradle-to-farm gate’ life cycle assessments.
	- *Food processing, marketing and distribution*: includes all functions that use primary food commodities and convert them to food products until they are sold at (super) markets. Food that the producer consumes herself or sells directly to the consumer does not pass the food processing and distribution sphere.
	- *Food preparation and consumption*: includes food preparation and ingestion as well as any transportation and storage occurring after the distribution stage.
	- *Residues management*: we refer here to food losses and wastes as food residues as long as they are still in a form that allows their valorization within (e.g. as fertilizer or packaging material) or outside the food system (e.g. to produce any other product). Residues management includes also management of non-organic residues (e.g. plastics, glass) that can be re-used or re-cycled.
	Food system spheres align with food system functions bundled in the stages as above with regard to their governance perspectives, but include further food system governance actors:
	- *Policy making:* including regional, national and international governments, conventions, and regulatory institutions
	- *Other actors:* including civil society, research & innovations, media & education, and public or private advisory systems
Food system boundaries*	Food system boundaries are defined by the food system functions that contribute to it. For non-global food system food system boundaries can be defined either from a territorial or consumption (diet) perspective:
	- *Diet food systems* are looking at all functions that contribute to the food value chains and residues management related with all food ingested by a certain group of people. This group can be defined by having similar preferences, same nationality, living in a specific area, working for a specific company, etc. Generally, a food producer, processors, … does not work exclusively for one single diet food system, but several diet food systems.
	- *Territorial food systems* are looking at all food system functions within a certain territory (e.g. country or region) as for diet food systems, but are including also all food functions in non-consumption food system stages that are serving other diet food systems (i.e. exporting to other countries/regions). Therefore, territorial food systems include also all food system functions for food that is imported to the territory and all food system functions happening in the territory, also if the goods are exported and consumed elsewhere. Territorial food systems are most relevant for policy making as they include all actors that can be reached directly or indirectly through food policies.

*New term as defined here.

**Common terms but without agreed definition.
